# Antibody-mediated stabilization of NRG1 induces behavioral and electrophysiological alterations in adult mice

**DOI:** 10.1038/s41598-018-26492-4

**Published:** 2018-05-29

**Authors:** Sara L. Dominguez, Ganapati V. Hegde, Jesse E. Hanson, Hong Xiang, Danielle Mandikian, C. Andrew Boswell, Cecilia Chiu, Yan Wu, Siao Ping Tsai, Daniel Fleck, Martin Weber, Hai Ngu, Kimberly Scearce-Levie, Erica L. Jackson

**Affiliations:** 1 Genentech, Neuroscience, South San Francisco, 94080 USA; 2 Genentech, Discovery Oncology, South San Francisco, 94080 USA; 3 Genentech, Developmental Sciences, South San Francisco, 94080 USA; 4 Genentech, Antibody Engineering, South San Francisco, 94080 USA; 5 Genentech, Biochemical and Cellular Oncology, South San Francisco, 94080 USA; 6 Genentech, Pathology, South San Francisco, 94080 USA; 7 Denali Therapeutics, Neuroscience, South San Francisco, 94080 USA; 8 ORIC Pharmaceuticals, Oncology, South San Francisco, CA 94010 USA

## Abstract

Neuregulin 1 (NRG1) is required for development of the central and peripheral nervous system and regulates neurotransmission in the adult. *NRG1* and the gene encoding its receptor, *ERBB4*, are risk genes for schizophrenia, although how alterations in these genes disrupt their function has not been fully established. Studies of knockout and transgenic mice have yielded conflicting results, with both gain and loss of function resulting in similar behavioral and electrophysiological phenotypes. Here, we used high affinity antibodies to NRG1 and ErbB4 to perturb the function of the endogenous proteins in adult mice. Treatment with NRG1 antibodies that block receptor binding caused behavioral alterations associated with schizophrenia, including, hyper-locomotion and impaired pre-pulse inhibition of startle (PPI). Electrophysiological analysis of brain slices from anti-NRG1 treated mice revealed reduced synaptic transmission and enhanced paired-pulse facilitation. In contrast, mice treated with more potent ErbB4 function blocking antibodies did not display behavioral alterations, suggesting a receptor independent mechanism of the anti-NRG1-induced phenotypes. We demonstrate that anti-NRG1 causes accumulation of the full-length transmembrane protein and increases phospho-cofilin levels, which has previously been linked to impaired synaptic transmission, indicating enhancement of non-canonical NRG1 signaling could mediate the CNS effects.

## Introduction

The *NRG1* gene encodes numerous isoforms of the Neuregulin 1 (NRG1) protein due to extensive alternative promoter usage and alternative splicing. All isoforms contain a common epidermal growth factor (EGF)-like domain that binds to and activates the ErbB3 and ErbB4 receptors. Most isoforms also contain a common cytoplasmic domain, whose function is not well characterized. Specific NRG1 isoforms are required for the proper development of the central and peripheral nervous systems^1,2^. NRG1 is also important for neurotransmission and synaptic plasticity in the mature nervous system, and signaling through its receptor ErbB4 has been shown to play a role in glutamatergic and GABAergic transmission in various brain regions^3^.

Numerous human genetic studies have identified *NRG1* as a schizophrenia susceptibility gene^4–8^. Schizophrenia is a severe neuropsychiatric disorder affecting ~1% of the general population worldwide. While current treatments can be effective in alleviating the positive symptoms, there is a major unmet need for treatments that impact the negative and cognitive symptoms. The causes of schizophrenia are multifactorial, including both genetic and environmental components^9,10^. Evidence for the contribution of pre- and perinatal factors to schizophrenia risk, and the occurrence of subtle structural changes in the brains of affected individuals indicate that defects in brain development contribute to schizophrenia pathology^11–13^. Genetic associations with the disorder have provided new insights into the molecular basis of schizophrenia. However, the presumed neurodevelopmental component makes it difficult to determine whether these factors continue to contribute to the disorder post-developmentally. Animal models that allow for reversible perturbations of the products of risk genes in the mature brain could facilitate the identification of targets for potentially disease-modifying therapies.

While genetic studies link *NRG1* and its receptor *ERBB4* to schizophrenia and other brain disorders, how NRG1 dysfunction impacts brain function is not fully understood. Many of the risk-associated SNPs occur in noncoding regions of NRG1, and could alter expression levels^14^, and both increases and decreases in NRG1/Erb4B levels and/or activity have been found in postmortem studies of schizophrenia patients^15–19^. Similarly, both genetically increasing and decreasing the levels of specific NRG1 isoforms in mice result in schizophrenia-like behaviors^4,20–23^. Importantly, turning on NRG1 overexpression in adult mice is sufficient to cause schizophrenia-like phenotypes that can be reversed by turning off NRG1 overexpression, suggesting an ongoing role for NRG1 dysfunction in adult pathophysiology^24^. In addition, abnormal cleavage of NRG1 could contribute to pathophysiology, as mice lacking NRG1 processing enzymes such as BACE1 and neuropsin show schizophrenia-like phenotypes^25,26^ and one of the few schizophrenia-associated SNPs known to occur in the coding sequence impairs cleavage of NRG1 by **γ**-secretase^27,28^. Whether the effects of disruption of NRG1 proteolytic processing are manifest during development or continue during adulthood, however, is unknown.

Here we report a novel means to perturb NRG1 signaling in the adult brain. We developed an anti-NRG1 antibody that blocks NRG1-receptor interaction and stabilizes full length transmembrane NRG1. Anti-NRG1 treated mice display motor abnormalities, as well as certain schizophrenia-like behavioral alterations, and impaired synaptic transmission. In addition, we show that anti-NRG1 treatment increases p-cofilin by modulating receptor-independent signaling. Thus, our study both provides new insight into the role of NRG1 on synaptic function and behavior and provides a new approach for *in vivo* modeling of certain schizophrenia-like phenotypes.

## Results

### Anti-NRG1 Induces Behavioral Alterations

**Figure 1:**
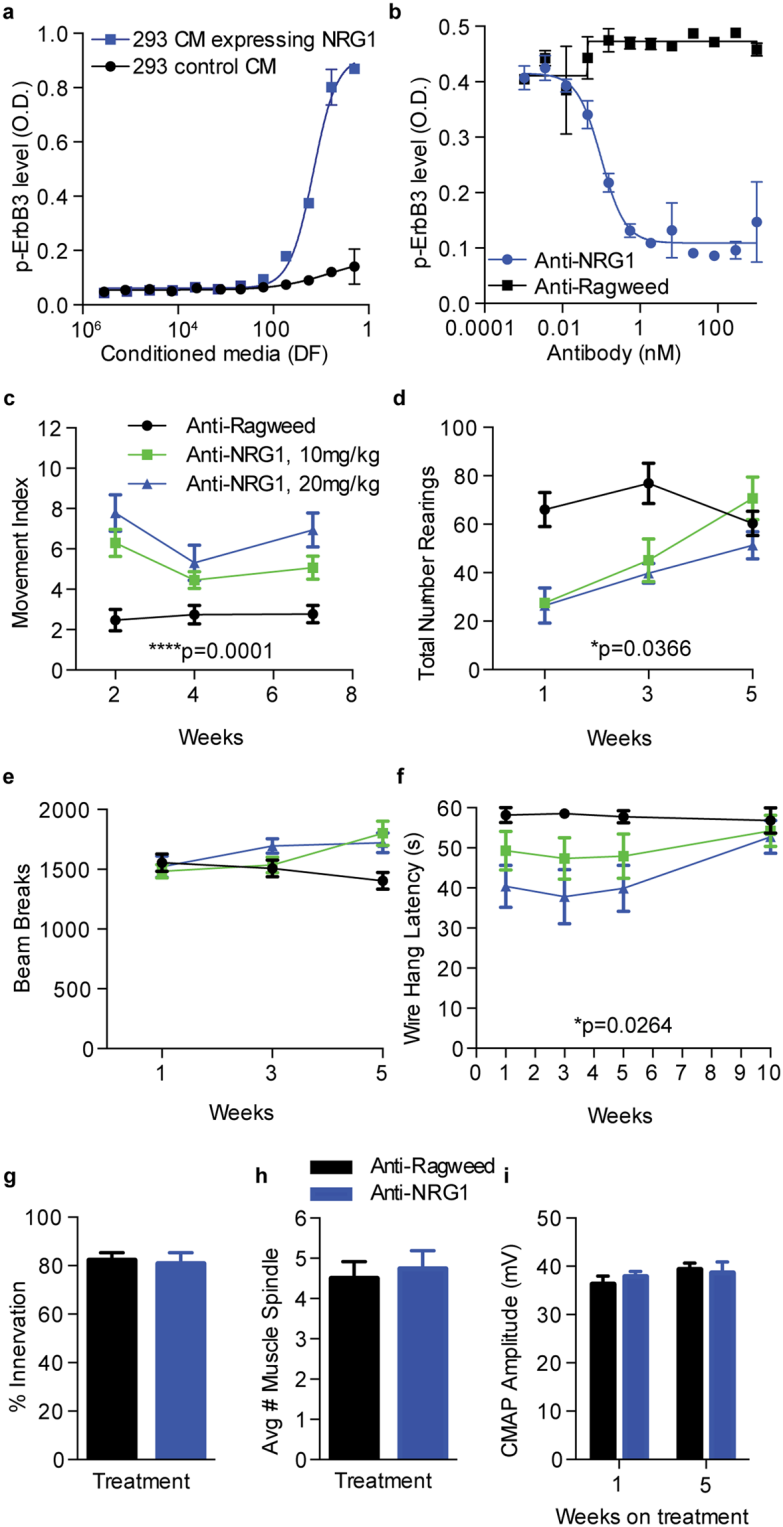
Treatment with mouse anti-NRG1 antibody causes changes in motor function. (**a)** Conditioned media containing mouse NRG1 significantly increases p-ErbB3 levels as determined by kinase receptor activation assay (KIRA). DF: dilution factor. (**b**) The cellular IC50 for anti-NRG1 against mouse NRG1 was determined by KIRA. (**c**) Dose dependent increase in movement index, measurements at 2, 4 and 7 weeks on treatment by mixed-model ANOVA with the factors treatment (F_(2,33)_ = 13.24; ****p < 0.0001) and time (F_(2,66)_ = 7.382; **p = 0.0013), and an interaction between treatment and time (F_(4,66)_ = 3.037; *p = 0.0232) (n = 12 F/group). (**d**) Number of rearings in open field by mixed-model ANOVA revealed a significant decrease in rearings by treatment group (F_(2,18)_ = 3.998; *p = 0.0366), across time (F_(2,36)_ = 13.51; ****p < 0.0001) and there is a treatment/time interaction (F_(4,36)_ = 5.294; **p = 0.0019) (n = 7 F/group). (**e**) Open field horizontal beam breaks by mixed-model ANOVA yielded only a time/treatment interaction (F_(4,66)_ = 5.024; **p = 0.0013) (n = 12 F/group) but no effect of treatment alone. (**f**) Wire hang test latencies by mixed-model ANOVA displayed significant decreases in hang time by treatment (F_(2,22)_ = 4.308; *p = 0.0264), across time (F_(3,33)_ = 3.570; *p = 0.0243) but no interaction between treatment and time. (**g**) Treatment with anti-NRG1 does not alter muscle synapse innervation after 5 weeks on treatment (p > 0.5) (n = 8 F/group). (**h**) Treatment with anti-NRG1 for 5 weeks does not alter the number of muscle spindles found in the gastrocnemius muscle (p > 0.05) (n = 7 F/group (6–8 sections per mouse). (**i**) Treatment with anti-NRG1 does not alter CMAP M wave amplitudes in the gastrocnemius muscle after 1 week or 5 weeks on treatment (p > 0.05) (n = 12 F/group). All data is shown as mean ± SEM.

We previously generated and characterized anti-NRG1 antibodies to explore their utility as cancer therapeutics^29,30^. While testing the efficacy of these antibodies in *in vivo* tumor models, we noticed that mice treated with anti-NRG1 exhibited hyperactivity and tremor. Therefore, we decided to conduct an in depth behavioral characterization of anti-NRG1-treated mice. Previously, fully human antibodies were generated against human NRG1 by phage display. To support chronic dosing in immune competent mice by reducing the risk of anti-therapeutic antibody response, the parental YW538.24.71 antibody was cloned onto the mouse IgG2 backbone. A cell-based ELISA assay measuring inhibition of ErbB3 phosphorylation upon stimulation with conditioned media from 293 cells overexpressing murine NRG1-beta1-ECD (Fig. 1a) was used to confirm activity of the antibody on the murine backbone. Anti-NRG1 showed potent, dose-dependent inhibition of ErbB3 phosphorylation, with an IC50 of 0.1 nM, demonstrating exquisite cross-reactivity with NRG1 (Fig. 1b).

To carefully characterize behaviors induced by anti-NRG1, we treated mice systemically with either 10 or 20 mg/kg of anti-NRG1. Injections were performed once weekly, for 10 weeks, and behavior data was collected from a battery of tests. Within the first week, an obvious full body tremor was visible in the anti-NRG1 treated mice. To quantify the movement induced by the tremor in an unbiased, automated manner, a mechanical reading of non-stimulated motor activity inside of a tight enclosure, that limits the ability for gross motor activity, was measured using a piezoelectric accelerometer. After 2, 4 and 7 weeks of antibody treatment, significant and dose-dependent increases in tremor-associated movement intensities, referred to as the “movement index”, were observed for the anti-NRG1 treatment groups in comparison with the control group (anti-Ragweed) (Fig. 1c). Next we tested if locomotor and/or exploratory activity were affected using the open field test. In this test, anti-NRG1 treated mice exhibited significantly fewer vertical rearings, when compared to controls after 1 or 3 weeks of dosing (Fig. 1d). Interestingly, by 5 weeks on treatment, all groups displayed similar numbers of rearings. Furthermore, horizontal locomotor activity was unaffected at these same 1 and 3 week time points (Fig. 1e), suggesting that anti-NRG1 treatment’s reduction in rearings was not due to an overall decrease in locomotion. To evaluate if changes in rearing were related to changes in limb strength, we subjected the mice to a wire hang test. Mice treated with anti-NRG1 had significantly shorter hang latencies than anti-Ragweed treated animals (Fig. 1f). This wire hang deficit also recovered after the 5^th^ week of dosing. Importantly, anti-NRG1 treatment had no effect on body weight (Supplementary Fig. S1) indicating that changes in body weight cannot account for the changes in wire-hang latencies observed.

Since neuregulin has been shown to be required for maintenance of neuromuscular function^31^, we sought to determine if anti-NRG1 treatment altered either the neuromuscular junction itself, or muscle spindles in the gastrocnemius muscle. We found no signs of denervation of the neuromuscular junction (NMJ) and no overall differences in muscle spindle counts in anti-NRG1 treated mice (Fig. 1g,h). Moreover, compound muscle action potential (CMAP) recordings showed no alterations in amplitude upon anti-NRG1 treatment (Fig. 1i), suggesting that overall muscle function is not being dramatically altered. However, the fact that there is no robust change in muscle architecture does not exclude the possibility that these motor deficits are caused by altered neurotransmission. In addition, it is also possible that the tremor present in the anti-NRG1 treated mice could affect both rearings and wire hang latency by impacting balance.

### Behavioral phenotypes caused by anti-NRG1 treatment are not present following anti-ErbB4 treatment

**Figure 2:**
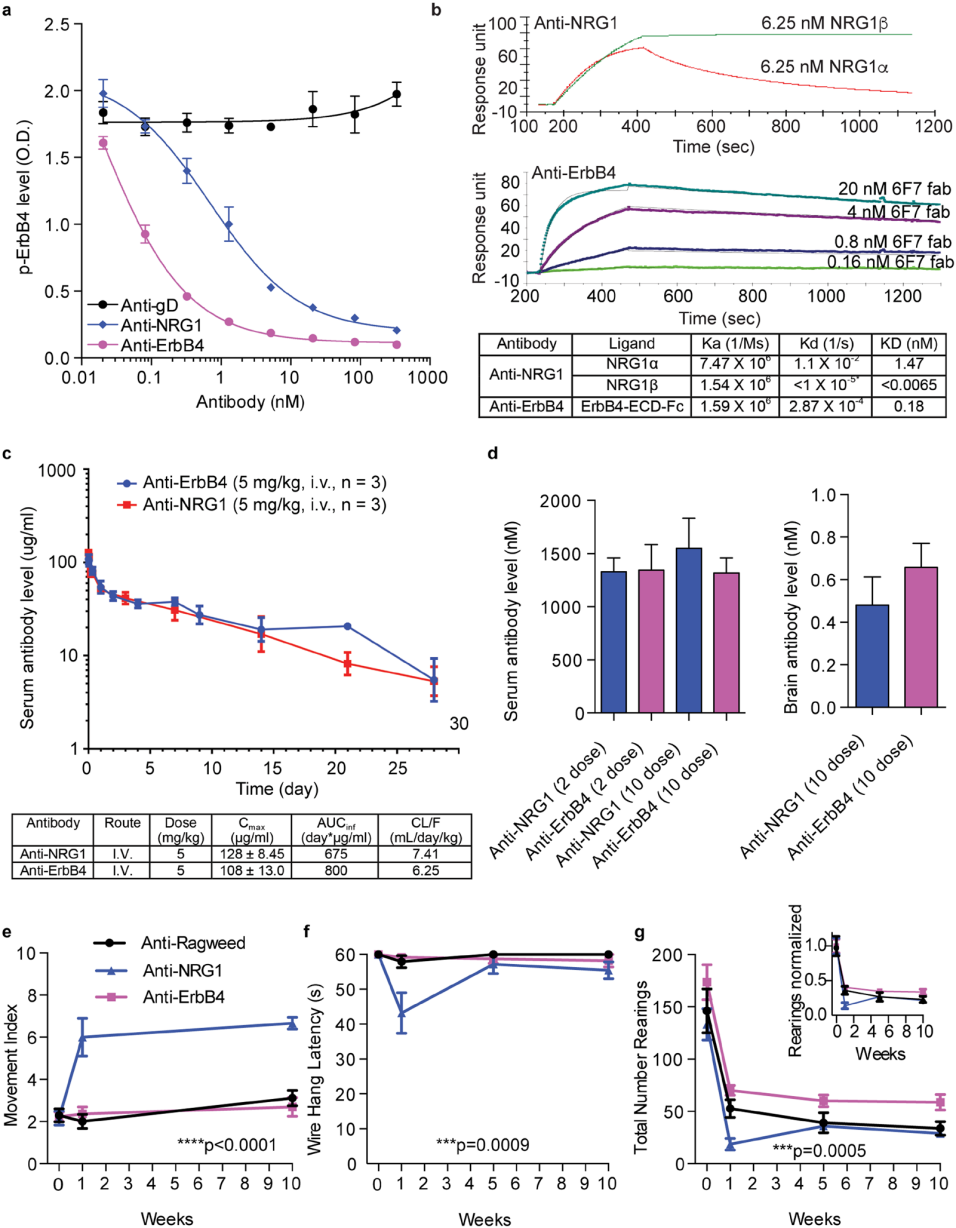
Anti-NRG1 but not anti-ErbB4 induces behavioral alterations despite similar blocking activity and PK properties. (**a**) Cellular IC50s for inhibition of NRG1-induced ErbB4 phosphorylation by anti-NRG1 and anti-ErbB4 were by KIRA with Anti-gD used as a control. (**b**) Affinity of anti-NRG1 and anti-ErbB4 antibodies by Biacore. *Kd of anti-NRG1 for NRG1β was below the detection limit of 10–5/s. (**c**) Single dose PK profiles of anti-NRG1 and anti-ErbB4 dosed at 5 mg/kg, IV in C57B6 mice. (**d**) Similar levels of Anti-NRG1 and anti-ErbB4 antibodies are detected in sera after 2 and 10 weekly doses of antibody and in brain after 10 weekly doses of antibody. Data is shown as mean ± SEM (n = 5 F/group). (**e**) Movement index measurements. Mixed-model ANOVA with the factors treatment and time revealed a significant increase in tremor across treatment groups (F(2,33) = 29.15; p < 0.0001) (n = 12 F/group). Post hoc comparisons found significant increases in tremor-associated movement intensities between Anti-NRG1 and Anti-Ragweed (****p < 0.0001) and between Anti-NRG1 and Anti-ErbB4 (****p < 0.0001) but no differences between Anti-ErbB4 and Anti-Ragweed. (**f**) Wire hang latencies. Mixed-model ANOVA yielded significant decreases in latencies across treatment groups F(2,33) = 8.696; p = 0.0009) (n = 12 F/group). Significant decreases in latency between Anti-NRG1 and Anti-Ragweed (**p < 0.01 Post hoc). (**g**) Number of open field rearings. Mixed-model ANOVA revealed a significant difference between treatments (F(2,23) = 10.63; p = 0.0005). Increased rearings were observed when comparing Anti-Ragweed to the Anti-ErbB4 group (*p < 0.05 Post hoc comparisons by Tukey’s t-test), but were not balanced predose. The inset is a percentage normalized for predose rearings to better depict rearing differences between dose groups. All data is shown as mean ± SEM and all post hoc comparisons by Tukey’s t-test.

In the nervous system, NRG1 is thought to signal mainly through the ErbB4 receptor, and both *NRG1* and *ERBB4* have been genetically linked to schizophrenia. Therefore, to better understand the mechanism of anti-NRG1-induced behavioral alterations, we next explored whether inhibition of ErbB4 signaling using anti-ErbB4 antibodies would cause similar phenotypes. In order to determine a comparable efficacious dose, we compared the potency of anti-NRG1 and anti-ErbB4 in inhibiting NRG1-induced phosphorylation of ErbB4 (Fig. 2a). Anti-ErbB4 more potently inhibited ErbB4 signaling in this assay (IC_50_ = 0.08 nM) than anti-NRG1 (IC_50_ = 0.8 nM). In addition, we determined the *K*_D_ of anti-NRG1 and anti-ErbB4 antibodies and found that both anti-NRG1 and anti-ErbB4 have high affinity against their targets, with anti-NRG1 having especially high affinity against NRG1-beta (Fig. 2b). We also compared the PK properties and brain exposure of the antibodies. Both antibodies had similar PK profiles in mice with clearance in the typical range for murine antibodies (Fig. 2c), and the trough brain concentrations of both antibodies at 6 days after the tenth dose were comparable (Fig. 2d). It is well established that ~0.1% of circulating antibodies cross the blood brain barrier (BBB)^32,33^. Brain levels of anti-NRG1 and anti-ErbB4 are ~0.03% and ~0.05% of circulating antibody levels respectively, and thus do not exhibit enhanced brain uptake. Even so, the mean trough brain concentration for anti-ErbB4, 0.6125 nM, was well above the IC_50_ for inhibition of NRG1-β-induced ErbB4 activation, while the mean trough brain concentration for anti-NRG1, 0.45 nM, was slightly lower than the IC_50_ for inhibition of NRG1-β induced ErbB4 activation.

If the behavioral changes caused by anti-NRG1 were dependent on disruption of signaling through the ErbB4 receptor, treatment with the high-affinity anti-ErbB4 molecule should have similar effects. In experiments comparing the effects of anti-NRG1 and anti-ErbB4 antibody treatment side by side, we again observed the described effects of anti-NRG1 treatment. However, we saw no tremor, or observable motor changes in anti-ErbB4 treated mice (Fig. 2e–g), indicating that the observed effects of anti-NRG1 likely did not depend on receptor mediated signaling.

To further explore whether the phenotypes observed in anti-NRG1 treated mice result from inhibition of receptor-mediated signaling, we treated with a combination of anti-ErbB4 and anti-ErbB3 antibodies, using a potent mouse cross-reactive ErbB3 blocking antibody. Once again, we found that anti-NRG1, but not anti-ErbB3/anti-ErbB4 combination treatment caused tremor and a decrease in rearings (Supplementary Fig. S2).

Notably, we did not detect changes in phospho-ErbB4 levels in the hippocampus of anti-NRG1 treated mice and phospho-ErbB3 levels were below the limit of detection by western blot (Supplementary Fig. S3), supporting a receptor-independent mechanism underlying the behavioral alterations. Since anti-ErbB4 has greater potency and an equivalent *in vivo* concentration, it is plausible to conclude that the observed differential behavioral effects for anti-NRG1 and anti-ErbB4 are not due to differences in antibody-mediated block of NRG1-ErbB4 signaling but instead result from a distinct effect of the anti-NRG1 antibody itself.

### Anti-NRG1 but not Anti-ErbB4 induces changes in CNS mediated behaviors

**Figure 3:**
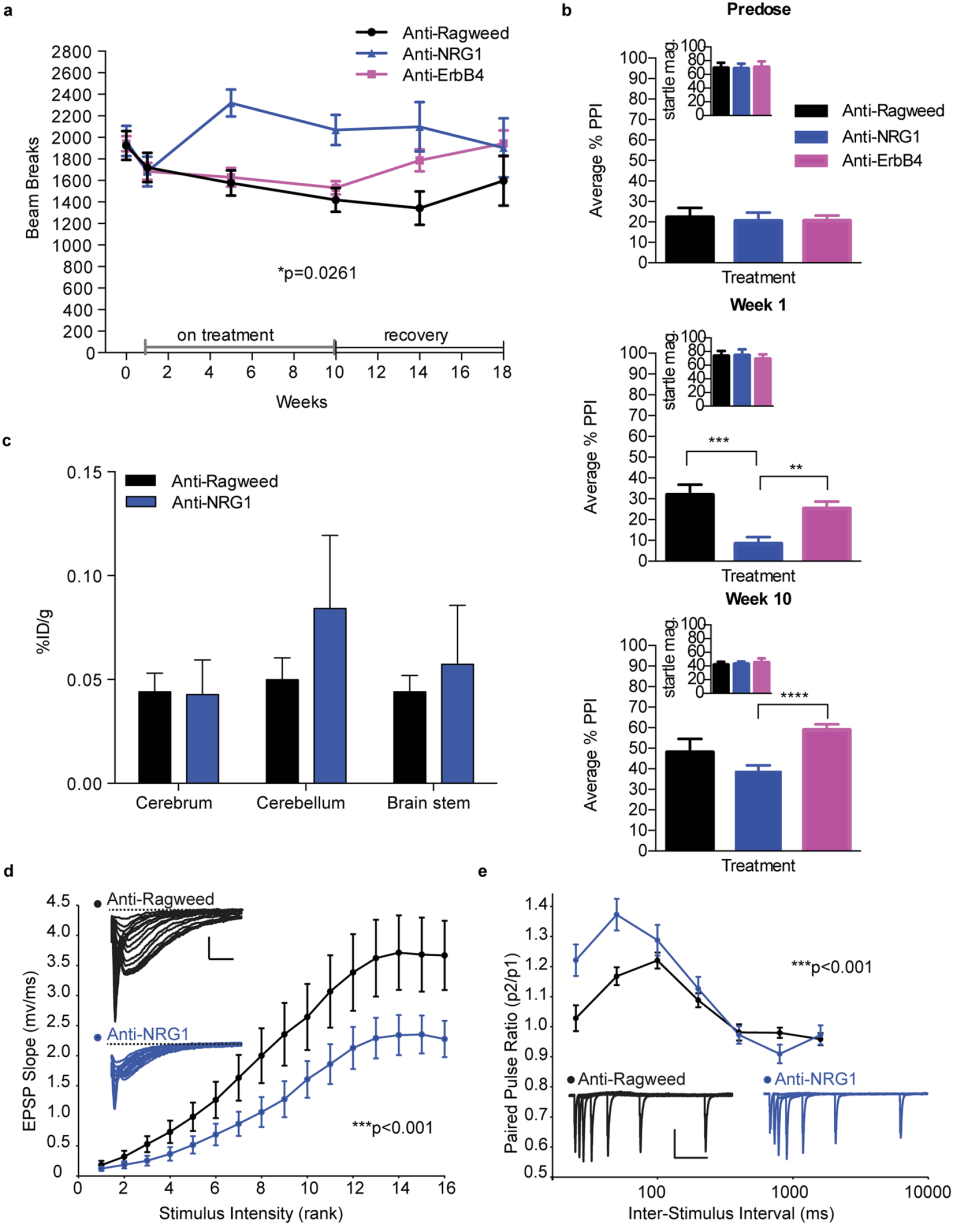
Anti-NRG1 induces CNS mediated behavioral alterations and synaptic impairment. (**a**) Number of beam breaks in open field. Mixed-model ANOVA with factors treatment and time revealed significant effects of treatment (F(2,22) = 4.323; p = 0.0261), and a treatment/time interaction (F(8,88) = 2.143; p = 0.0399) (n = 7–11 F/group). Post hoc with Tukey’s multiple comparisons test showed significantly more beam breaks with Anti-NRG1 vs Anti-Ragweed (*p < 0.05). (**b)** PPI averaged across multiple prepulse intensities (n = 12 F/group). **Predose:** No difference where observed between treatment groups prior to treatment. **Week 1:** One way ANOVA reveals significant effects of treatment (F(2,33) = 9.683; p = 0.0005) with post hoc comparisons, t-test showing a treatment effect between anti-NRG1 vs. anti-Ragweed (***p = 0.0006) and also with anti-NRG1 vs. anti-ErbB4 (*p = 0.0013). **Week 10:** One way ANOVA again revealed an effect of treatment (F(2,33) = 5.135; p = 0.0113) with post hoc comparisons only showing a significant effect between Anti-NRG1 and anti-ErbB4 (****p < 0.0001). (**c**) *In vivo* biodistribution of 111In-labeled NRG1 in the presence of anti-NRG1 or anti-ragweed antibodies. Blood-corrected data presented as percent of injected dose normalized to tissue weight (%ID/g) (n = 5/group). (**d**) Synaptic function evaluated in brain slices by measuring EPSP slope in response to different stimulus intensities (n = 17 slices/mouse and 5 mice/group). Example EPSPs shown in inset (scale bars, 1 mV and 1 ms). Significant effect of stimulus intensity (F(1,528) = 43.0, ***p < 0.001), and treatment on EPSP slope (F(15,528) = 17.7, ***p < 0.001). (**e**) Paired pulse ratio at different inter-stimulus intervals (n = 18 slices/mouse and 5 mice/group). Example EPSPs are shown inset (scale bars are 0.4 mV and 200 ms). Significant effects of interval (F(1,252) = 10.3, p = 0.002), and treatment (F(6,252) = 27.4, p < 0.001) and a significant interaction between treatment and interval (F(6,252) = 3.9, p < 0.001). Post hoc analysis showed significant effects of treatment within 25 ms (p < 0.001) and 50 ms intervals (p < 0.001). All data is shown as mean ± SEM.

Some aspects of the behavioral alterations induced by anti-NRG1 treatment suggest possible CNS effects. Though initial activity levels in the first two weeks remained similar between dose groups, open field assessment revealed additional changes in horizontal locomotor activity beginning at week 5 of dosing in the anti-NRG1 but not significantly in anti-ErbB4 treated mice (Fig. 3a). As dosing continued, the anti-NRG1-treated mice developed a significant and long lasting hyperactivity (Fig. 3a, Supplementary Fig S4).

To better understand whether certain behavioral changes caused by anti-NRG1 treatment could be a result of altered signaling in the CNS, we evaluated behaviors more directly related to CNS changes and/or schizophrenia itself. First, we looked for disruption of pre-pulse inhibition (PPI) of the acoustic startle response, a behavior that is altered both in human schizophrenic patients and several putative rodent models of schizophrenia. After 1 week of treatment, we observed a significant decrease in PPI in the anti-NRG1 treated mice, while PPI in anti-ErbB4 was not significantly altered (Fig. 3b). A trend towards decreased PPI was still apparent at 10 weeks on treatment (Fig. 3b, week 10) when levels of PPI were higher in all treatment groups. Importantly, the deficits in PPI were independent of any changes in general startle responsiveness, as treatment had no effect on startle magnitude (insets, Fig. 3b).

To further explore whether the PPI deficits observed in the anti-NRG1 treated mice are receptor independent, we evaluated PPI in animals treated with the anti-ErbB4/anti-ErbB3 combination. Again we found that after 1 week of antibody treatment anti-NRG1 treated mice, but not anti-ErbB4 or anti-ErbB4/ErbB3 treated mice, showed significant decreases in PPI (Supplementary Fig S5).

### Anti-NRG1 does not cause peripheral sequestration of NRG1

It has been reported that NRG1 passes the blood brain barrier and activates ErbB4 in the brain^34,35^. Therefore, a possible explanation for the potential CNS effects of anti-NRG1 is that anti-NRG1 may sequester circulating NRG1 in the periphery, preventing it from entering the brain and activating receptor-mediated signaling. To evaluate the effects of anti-NRG1 on NRG1 biodistribution, we labeled exogenous NRG1 with a radioactive tracer (^111^In) and assessed its pharmacokinetics (PK) and biodistribution in the presence or absence of anti-NRG1. Animals were dosed with either anti-NRG1 or anti-ragweed and one hour later, all mice were dosed with tracer amounts of ^111^In-NRG1. Blood samples were collected over the course of the following hour and the PK of free NRG1 and NRG1:antibody complexes were determined. One hour after tracer dosing, animals were euthanized and tissues collected and analyzed for radioactivity. In brain there were low but similar amounts of ^111^In-NRG1 and/or ^111^In-NRG1:anti-NRG1 complex in both groups (Fig. 3c), indicating that anti-NRG1 does not significantly affect brain uptake of exogenously administered NRG1. Together, these results suggest that anti-NRG1 is not inducing peripheral sequestration of NRG1.

### Anti-NRG1 treatment alters synaptic physiology

In order to directly evaluate the effects of anti-NRG1 on CNS function, we assessed several synaptic parameters in a subset of the behavior-tested mice after 10 weeks of treatment. Synaptic strength was examined by assessing the input-output relationship of Schaffer collateral synapses onto CA1 pyramidal neurons using field EPSP recordings in brain slices. This experiment showed a significant reduction in synaptic strength in the anti-NRG1 treated animals compared to the anti-Ragweed treated controls (Fig. 3d). Next the response to paired-pulse stimulation was measured, which results in facilitation at shorter inter-stimulus intervals. Anti-NRG1 treated animals exhibited significantly enhanced paired pulse facilitation (Fig. 3e). As the magnitude of such paired pulse facilitation is generally believed to be inversely proportionate to presynaptic release probability, this suggests a lower basal presynaptic release probability in the anti-NRG1 treated mice. Together, these results indicate that anti-NRG1 antibodies directly alter brain function and behavior by inducing synaptic dysfunction.

### Anti-NRG1 treatment enhances non-canonical signaling

**Figure 4:**
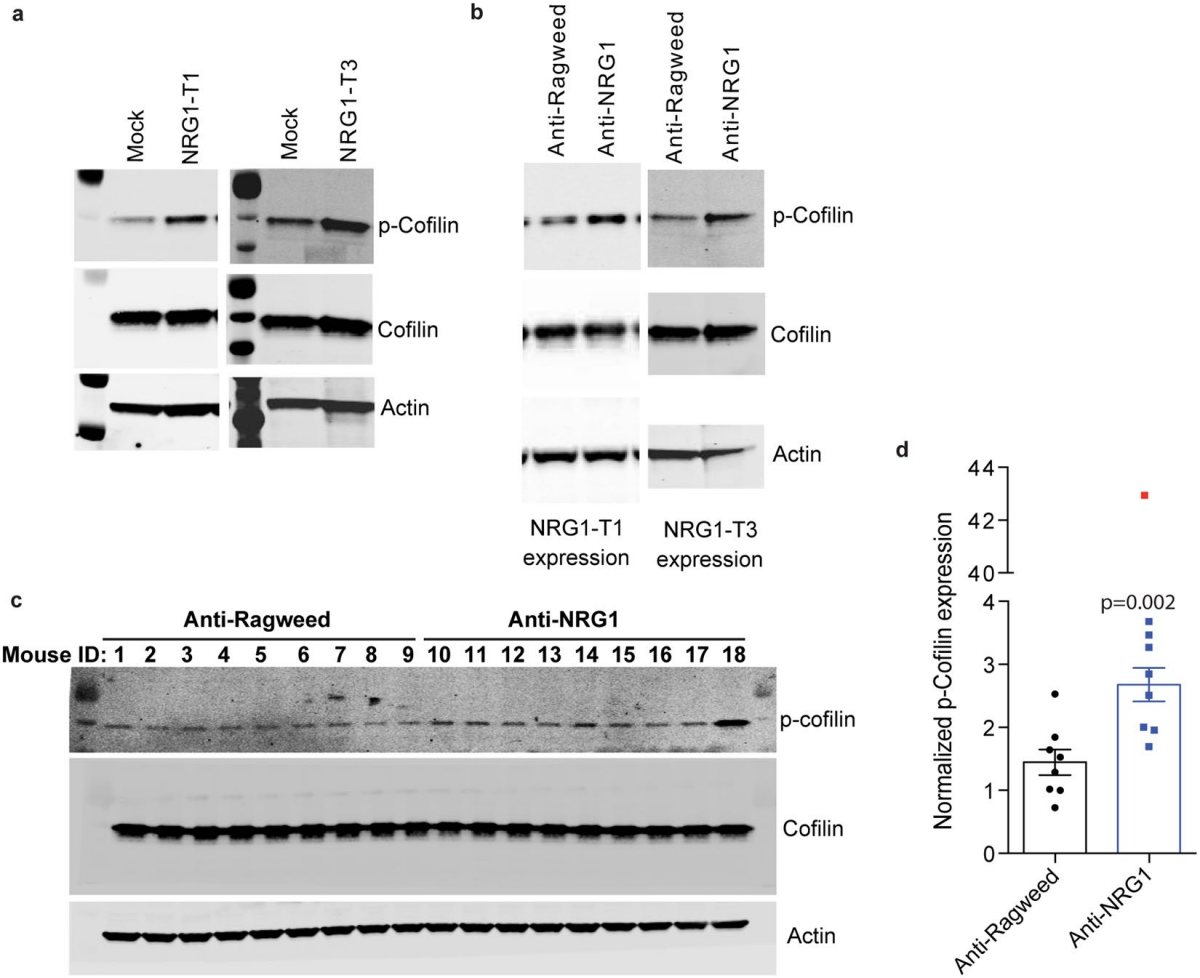
Induction of Cofilin phosphorylation by anti-NRG1 is receptor independent. (**a**–**c**) Western blots showing increased p-cofilin levels in (**a**) 293 cells transiently transfected with NRG1 types I and III. (**b**) Transiently transfected cells treated with anti-NRG1. (**c**) Lysates of hippocampus from conditional ErbB4^−/−^ mice treated with anti-NRG1. (**d**) Significant increase in normalized p-cofilin expression upon anti-NRG1 treatment in ErbB4^−/−^ mice as quantified from western blot. (p = 0.002 unpaired t-test). The data point indicated in red is considered an outlier, and excluded from calculation of mean and statistical analysis.

In addition to canonical signaling, NRG1 can participate in non-canonical signaling which can be receptor-independent^36–38^. The behavioral and synaptic deficits in the anti-NRG1 treated mice were strikingly similar to those recently described by Yin *et al*. upon overexpression of NRG1 in forebrain excitatory neurons^24^. Synaptic impairment in this system was linked to non-canonical NRG1 signaling and involved increased phospho-cofilin levels at synapses. The ability to reorganize actin is critical for synaptic function^39^ and phosphorylation of cofilin inactivates its actin severing abilities, impairing actin dynamics. If anti-NRG1 were altering synaptic function and behavior by a similar mechanism, we would expect that anti-NRG1 treatment would also increase phospho-cofilin levels. To evaluate this, we transfected 293 cells with full length NRG1 type I and III and analyzed phospho-cofilin levels in the presence and absence of anti-NRG1. Phospho-cofilin levels increased upon transfection of NRG1 and levels were further increased by anti-NRG1-treatment (Fig. 4a,b). Next, we sought to determine whether NRG1 antibodies cause a receptor-independent increase in phospho-cofilin levels in the brain. To test this hypothesis, we dosed brain-specific ErbB4−/− mice with anti-NRG1 and determined the levels of phospho-cofilin in the hippocampus. We observed a significant increase in phospho-cofilin in the anti-NRG1 treated mice (Fig. 4c,d). Together with the lack of behavioral changes in anti-ErbB4-treated mice, these data strongly suggest that non-canonical signaling through phospho-cofilin, rather than ErbB4 receptor-dependent signaling, mediates the anti-NRG1-induced behavioral and electrophysiological alterations.

### Anti-NRG1 treatment causes accumulation of full length NRG1

**Figure 5:**
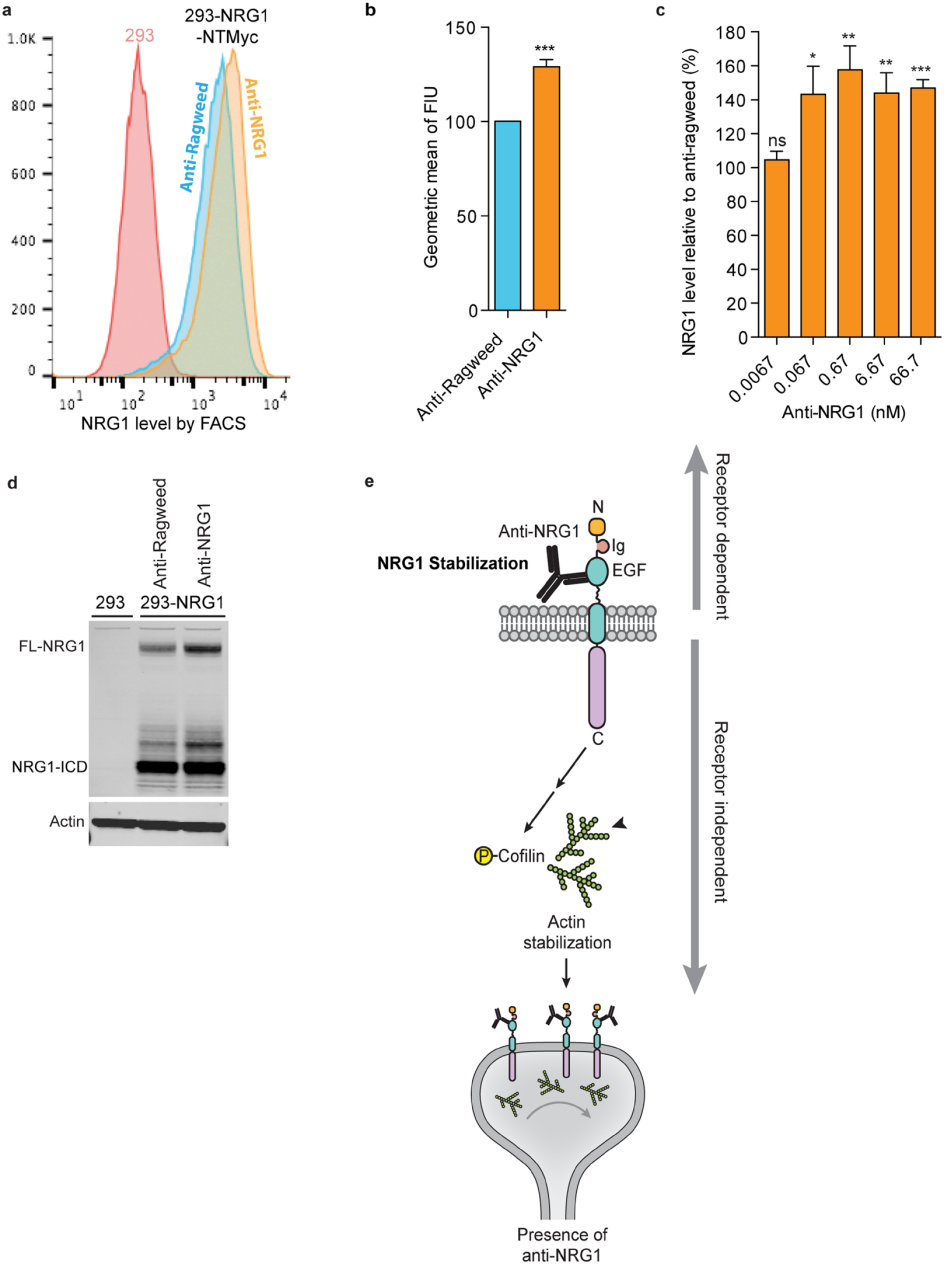
Anti-NRG1 causes accumulation of full length NRG1. (**a**) Representative FACS profile showing an increase in full length NRG1 in 293 cells stably expressing NRG1-NT-Myc upon treatment with anti-NRG1 relative to control anti-Ragweed antibody. (**b**) Significant increase in full length NRG1 upon anti-NRG1 treatment as determined by FACS using 293 cells stably expressing NRG1-NT-Myc. Data is presented as geometric mean of fluorescence intensity unit (FIU) normalized to the mean anti-Ragweed signal. (**c**) Significant increase in full length NRG1 in presence of anti-NRG1 as determined by ELISA. Data is mean ± SEM from 3 independent experiments. *p < 0.05, **p < 0.001, ***p < 0.0001 compared to respective concentration of anti-Ragweed. (**d**) Representative western blot showing an increase in full length NRG1 upon treatment with anti-NRG1. All data are shown as mean ± SEM. (**e**) Model of proposed mechanism of anti-NRG1-mediated changes. NRG1 is a transmembrane protein. Anti-NRG1 binds to the EGF like domain on the extracellular portion of membrane bound NRG1 blocking receptor binding and inhibiting receptor-mediated signaling (receptor dependent signaling). Binding of anti-NRG1 to the EGF-like domain also increases levels of full length NRG1. In turn, the intracellular domain of membrane-bound NRG1 induces phosphorylation of Cofilin. P-Cofilin is inactive in function leading to actin stabilization, which has been implicated to impair glutamate release causing synaptic deficits (receptor independent NRG1 signaling).

Given the similarity to changes seen in NRG1 overexpressing mice, and the fact that brain levels of anti-NRG1 are well above its *K*_D_ for NRG1 binding, we hypothesized that the anti-NRG1/NRG1 interaction may cause stabilization and accumulation of full length NRG1. To test this hypothesis, we treated 293 cells stably overexpressing N-terminally Myc-tagged NRG1 (NRG1-NTMyc) with anti-Ragweed or anti-NRG1 and determined the levels of full length NRG1 (FL-NRG1). FACS analysis of live 293-NRG1-NTMyc cells stained with anti-Myc antibody showed an increase in mean fluorescence intensity of cells treated with anti-NRG1 relative to control antibody (Fig. 5a,b). A similar increase in FL-NRG1-NTMyc levels was found using a cell based ELISA assay (Fig. 5c). Furthermore, we corroborated this finding by western blot. There was a robust increase of FL-NRG1 when 293 cells stably expressing NRG1 were cultured with anti-NRG1 compared to anti-Ragweed (Fig. 5d). While there was no apparent change in the intensity of NRG1-ICD band, the ratio of FL-NRG1 to NRG1-ICD was increased, and these changes were similar to those observed in cells treated with a cocktail of sheddase inhibitors (Supplementary Fig. S6).

Overall, our results indicate that stabilization of NRG1 by anti-NRG1 activates receptor-independent signaling leading to increased phosphorylation of cofilin, synaptic dysfunction, and schizophrenia-relevant behavioral changes in mice (Fig. 5e).

## Discussion

In this study, we used a novel antibody-based approach to modulate the function of endogenously produced NRG1 in adult mice. We developed an antibody that both blocks NRG1-receptor interaction, and stabilizes full-length transmembrane NRG1. Anti-NRG1 treated mice display behavioral changes that include tremor, recoverable alterations in motor function, as well as hyperlocomotion and impaired sensorimotor gating, which are both reported phenotypes in animal models of schizophrenia. Electrophysiology in hippocampal slices from chronic anti-NRG1 treated mice revealed reduced synaptic transmission and enhanced paired-pulse facilitation, consistent with decreased glutamatergic function, which has also been implicated in schizophrenia^40^.

Multiple risk haplotypes and genetic polymorphisms in both the 5′ and 3′ regions of the NRG1 gene have been linked to schizophrenia^41^ Variants linked to schizophrenia have been shown to alter transcription factor binding and to increase transcription of specific NRG1 isoforms, and have been associated with abnormal cortical function and psychotic symptoms^14,42^. Moreover, elevated levels of multiple NRG1 mRNA isoforms have been detected in the blood of clozapine-treated schizophrenia patients, with more highly elevated levels frequently detected in those with an earlier age of illness onset^43^.

Interestingly, the ratio of proteolytically processed to full length NRG1 is decreased in Brodmann’s area 6 of schizophrenia patients, suggesting that proteolytic processing of NRG1 is impaired in schizophrenia^44^. One of the few SNPs known to occur in the coding sequence of NRG1 is a Val-to-Leu mutation in the transmembrane domain. This substitution impairs cleavage of NRG1 by γ-secretase^27,28^. Regulated intramembrane proteolysis is typically a stepwise process, with γ-secretase cleavage occurring only after sheddase cleavage of the ectodomain^45^. We observe a notable increase in full length NRG1 protein upon antibody treatment, and a change in the ratio of full length to processed NRG1. Therefore, it is tempting to speculate that the observation of schizophrenia-related phenotypes after anti-NRG1 dosing is caused by antibody-mediated inhibition of full length NRG1 shedding. Although further work is required to establish this mechanism, such impaired shedding would subsequently lead to a reduction in γ-secretase cleavage thereby linking our animal model to the human disorder.

It was recently shown that inducible overexpression of NRG1 in CAMK2a+ excitatory neurons results in impaired glutamatergic transmission and schizophrenia-related behavioral alterations, which are reversed when the NRG1 transgene is turned off. The glutamatergic hypofunction is ErbB4-independent and LIMK-dependent, presumably mediated by an interaction between LIMK and the NRG1 intracellular domain. Because cell autonomous effects of signaling from the NRG1 ICD underlie the phenotype, results of this and earlier overexpression studies could have depended on the cell type(s) in which NRG1 was overexpressed. We demonstrate that anti-NRG1 treatment increases p-Cofilin levels in cells expressing the NRG1 type III isoform, the most abundant isoform in the brain. Since anti-NRG1 causes accumulation of NRG1 on cells endogenously expressing NRG1, our work corroborates and expands on these findings.

Sustained tremor was a striking early-onset phenotype in the anti-NRG1 treated mice. Tremor was not observed in the mice overexpressing NRG1 in CAMK2a-expressing neurons, but was reported in Thy-1-NRG1 transgenic mice^23,46^. Our findings suggest that the lack of tremor in CAMK2a-regulated model is due to the restriction of NRG1 overexpression to a particular cell type. While earlier studies attributed the tremor in Thy-1-NRG1 mice to hypermyelination, the rapid onset of tremor in the anti-NRG1-treated mice suggests that NRG1 accumulation induces tremor in a manner that is independent of its effects on myelination, it is conceivable that altered neurotransmission could also contribute to this phenotype. Interestingly, movement disorders including tremor, were reported in schizophrenia patients long before the advent of antipsychotics, thus likely reflecting a true manifestation of the disease^47^. However, because motor abnormalities are also a side effect of antipsychotic drugs, they may go unrecognized as intrinsic disease pathobiology^48^. Reports on the prevalence of tremor in treatment-naive schizophrenia vary, with rates based on mechanical measurements reaching as high as 37% of patients^49,50^. Tremor has also been found to occur in 27% of non-psychotic siblings of patients with nonaffective psychosis, suggesting it may be linked to the genetic causes of the illness^51^. Our findings demonstrate that tremor is caused by anti-NRG1 treatment, possibly via induced stabilization of transmembrane NRG1, suggesting it may be linked to certain genetic drivers of the disorder.

Both NRG1 and its receptor ErbB4 have been genetically linked to schizophrenia, and ErbB4 deficient mice display some schizophrenia-related phenotypes. The anti-NRG1 antibody used in this study both stabilizes full length NRG1 and blocks NRG1-mediated ErbB4 activation. Thus, both increased signaling from the NRG1 ICD and reduced ErbB4 activity could contribute to the observed phenotypes. However, we did not observe any behavioral alterations in mice treated with an anti-ErbB4 antibody that was more potent in inhibiting NRG1-mediated ErbB4 activation. Moreover, treatment with a combination of both anti-ErbB3 and anti-ErbB4 inhibitory antibodies did not result in tremor or sensorimotor gating deficits, further supporting a receptor-independent mechanism of the anti-NRG1 phenotypes. In addition to validating the findings reported by Yin *et al*.^24^ that receptor-independent NRG1 signaling causes glutamatergic hypofunction, our work indicates that the behavioral alterations are also receptor-independent and suggests that the phenotypes displayed in ErbB4 mutant mice could be developmental in origin.

In addition to providing insight into the role of NRG1 processing in CNS function, our results describe a novel model with relevant phenotypes for studying pathophysiological processes of schizophrenia. Using administration of anti-NRG1 to cause phenotypes with relevance to schizophrenia is attractive because it allows for temporal control, which may help to distinguish whether the alterations in *NRG1* associated with schizophrenia cause abnormal neurodevelopment or perturb NRG1 signaling required for proper brain function in the adult. Furthermore, in contrast to transgenic overexpression, anti-NRG1 modulates endogenous NRG1 without making any assumptions regarding appropriate cell types or NRG1 isoforms, thus making it more relevant to address mechanistic questions and test therapeutic hypotheses. Anti-NRG1 can be easily administered in the context of existing transgenic or knockout mice in order to study the multi-genic basis of the disorder. As such, this approach provides a powerful new tool for exploring the multifactorial basis for psychiatric disorders involving NRG1 dysfunction.

## Methods

### Antibody characterization

NRG1, ErbB4 and Ragweed antibodies were developed at Genentech as described^29^. Conditioned media from 293 cells expressing the murine NRG1b EGF-like domain was concentrated using centricon tubes (Millipore). Cross reactivity of anti-NRG1 to mouse NRG1 was determined by KIRA as previously described^29,52^. Cellular IC50 of NRG1 and ErbB4 antibodies was determined as follows. Briefly, serum-starved H522 cells cultured in 96-well plates were treated with serial dilutions of antibodies for 30 minutes followed by treatment with a fixed amount of NRG1-beta-ECD for 30 minutes at 37 °C in a CO_2_ incubator. After decanting the medium, cells were lysed and p-ErbB3 levels were measured by ELISA.

### Antibody binding affinities

Antibody binding affinities and rate constants were measured by Surface Plasmon Resonance (SRP) using a BIAcore™-T200 instrument. The kinetic parameters were determined via antibodies directly coated on the CM5 biosensor chips to achieve approximately 800 RU. Four-fold serial dilutions (100 nM to 0.024 nM) of ligands (ErbB4-ECD-Fc, NRG1α, and NRG1β) were then injected in HBS-T at 25 °C with a flow rate of 30 μl/min. Association rates (*k*_on_) and dissociation rates (*k*_off_) were calculated using a simple one-to-one Langmuir binding model (BIAcore Evaluation T200 Software version 2.0). The equilibrium dissociation constant (*K*_D_) was calculated as the ratio *k*_off_/*k*_on_. For affinity analysis, *K*_D_ was calculated using a steady state affinity model.

### Mice

C57BL/6 J mice were obtained from Jackson Labs (Bar Harbor, ME) and were housed on a regular light/dark cycle (14:10 hours) with *ad libitum* access to food and water. Behavioral assessments were conducted during the light phase. All protocols for mouse experiments were approved by the Genentech Institutional Animal Care and Use Committee and were conducted in accordance with the NIH Guide for the Care and Use of Laboratory Animals.

### Antibody formulation, and dosing

All antibodies were formulated in phosphate buffered saline (PBS). Mice were dosed weekly with intraperitoneal injections of anti-NRG1 (10 or 20 mg/kg), anti-ErbB4 (25 mg/kg) or anti-Ragweed (20 mg/kg) unless otherwise noted.

### Pharmacokinetic (PK) assessment of antibodies in serum and brain

At the specified times blood was collected by retro-orbital bleed followed by serum collection. Brains were collected and tissue was homogenized in 1% NP40 containing protease inhibitors (Roche), and the supernatant was collected for further analysis. Protein was measured by bicinchoninic acid (BCA) assay according to manufacturer’s instructions (Thermo Scientific). Antibody levels in sera and brain lysate were determined by enzyme-linked immonosorbent assay (ELISA).

### Behavioral tests

All scoring was done blind to treatment and testing was counter-balanced across time, recording chamber and between dose groups.

#### Open Field

Automated PAS Open Field recording systems (San Diego Instruments, San Diego, CA) were used to record spontaneous locomotor activity. Mice were placed individually in transparent Plexiglas chambers (40.5 (W) × 40.5 (L) × 38 (H) cm) and horizontal and vertical movements were recorded with two frames fitted with infrared beams. Activity was measured over 15 minutes by calculating the total number of beam breaks and rearings per session.

#### Wire Hang

Mice were placed on a wire cage lid, allowed to grasp the wires and the lid was gently inverted. The time the mouse was able to hang suspended was recorded, with a maximum latency of 60 seconds. Mice unable to reach the full 60 seconds could repeat up to 3 times, after which their maximal score was recorded.

#### Movement Index (tremor)

The movement index was measured and quantified in the startle chambers by averaging the overall, non-stimulated movement intensities during PPI testing.

#### Prepulse Inhibition of Acoustic Startle

Startle chamber specifics can be found in the supplemental methods

All prepulse inhibition (PPI) sessions included multiple *pulse-alone, nostim* (no-stimulus) and *prepulse* + *pulse* trials. *Pulse-alone* trials consisted of a 120 dB(A) broad-band noise, lasting 40 ms. *Nostim* trials consisted of only background noise (65 dB(A) and were interspersed between all active trials (pulse alone or prepulse + pulse trials). *Prepulse* + *pulse* trials consisted of a 20 ms noise burst at 4, 8, or 16 dB(A) above background followed by the pulse 100 ms later (onset to onset). Sessions began with a 5-min acclimation period where a background noise level of 65 dB(A) was presented and continued throughout the session. Following acclimation, 5 presentations of the *pulse-alone* trial occurred, after which, a succession of *prepulse* + *pulse* or *pulse alone* trials were presented in pseudorandom order. At the end of the session 55 more *pulse-alone* trials were presented. There was an average inter-trial interval (ITI) of 15 s (range: 8–22 s). *Nostim* trials were not included in the calculation of the ITIs. Total session duration was approximately 18.5 min. Startle response calculations can be found in the supplemental methods.

#### CMAP recordings

Mice were anesthetized with 2.5% isoflurane, shaved and two stimulating needle electrodes were inserted, perpendicular to the nerve into either side of the right sciatic notch. Recording needle electrodes were inserted into the Achilles tendon (anode) and the gastrocnemius muscle (cathode), and a digital ring ground electrode, coated in electrode cream, was placed on the mouse’s tail. Data was amplified (BioAmp, ADinstruments) and acquired with a sampling rate of 10 kHz, and filtered at 1 Hz high pass and 5 kHz low pass (Powerlab 4/25, ADInstruments). A controlled stimulus, with a pulse duration of 0.2 ms, was applied to the nerve to evoke contractions from the GA muscle in 2 mA increments, starting from 2 to as high as 50 mA, until the amplitude no longer increased. The maximum main (M) wave amplitude was recorded for each mouse.

### Muscle Histology

Mice were dosed for 5 weeks and euthanized 48 hours post final dose. Details on tissue collection, muscle sectioning and sampling can be found in supplemental materials. Immunoflurescent staining for neuro muscular junctions was conducted as described^53^ and muscle spindle staining and quantification were conducted as described^31^.

### Electrophysiology

Recordings were performed in oxygenated Artificial Cerebrospinal Fluid (ACSF) containing (in mM) 127 NaCl, 2.5 KCl, 1.3 MgSO4, 2.5 CaCl2, 1.25 Na2HPO4, 25 NaHCO3 and 25 mM glucose. 400-µm coronal hippocampal slices were prepared in ice-cold oxygenated ACSF with the MgSO4 concentration elevated to 7 mM, NaCl replaced with 110 mM choline and with 12 mM Na-ascorbate, and 3 mM Na-pyruvate added. Field EPSPs were measured from the stratum radiatum of CA1 in response to stimulation of Schaffer collateral inputs. Input-output relationships were measured by stimulating at logarithmically spaced stimulus intensities up 1000 mA. Paired pulse ratios were measured using stimuli separated by 25, 50, 100, 200, 400, 800 or 1600 ms. Significance was assessed using ANOVA and follow up analysis was performed using the Holm-Sidak method.

### Brain distribution of In-111 labeled NRG1

Dota conjugation and radiolabelling methods can be found in supplemental materials.

#### *In vivo* study design and analysis

Male C57BL/6 J mice were obtained from Jackson/West (CA) and two groups of 5 mice were used for this study. One group received an IV injection of anti-NRG1 antibody and the other received anti-ragweed, each at 20 mg/kg. After 1 hour, all mice received 5 µCi of [In-111]-DOTA-NRG1, diluted in PBS. At 10 and 30 minutes, all mice were bled retro-orbitally under isoflurane (inhalation to effect). At 1 hour, animals were euthanized under anesthesia of ketamine (75–80 mg/kg)/xylene (7.5–15 mg/kg) by thoracotomy. A final blood was drawn via cardiac puncture and tissues were collected, rinsed in cold PBS, blotted dry, weighed and frozen. Brain was divided into cerebrum, cerebellum and brain stem. Sample radioactivity was measured using a 1480 WIZARD Gamma Counter in the energy window for the 245 keV photon peak of ^111^In (t_1/2_ = 2.8 days) with automatic background and decay correction. Tissue distribution data was processed to correct for blood contamination as previously described^54^.

### Western blot analysis

For *in vitro* NRG1 processing experiments 293 cells were transfected with NRG1 type I or III and treated with 20 ug/ml antibodies for 72 hours. For evaluation of signaling in brains of anti-NRG1-treated mice, wild type or ErbB4^−/−^ mice were treated with anti-NRG1 or anti-ragweed (20 mg/kg, i.p., 2 dose). Treated cells from *in vitro* experiments of hippocampus of antibody treated mice were lysed in cold RIPA buffer containing protease and phosphatase inhibitor cocktails (Thermo Scientific) using homogenizer. Protein concentration was determined by BCA (Thermo Scientific). Proteins were separated by SDS-PAGE and transferred to nitrocellulose by iBlot. Primary antibodies used were anti-actin (BD Biosciences), anti-NRG1, anti-ErbB3 and anti-ErbB4 (Santa Cruz Biotechnology), anti-phospho-ErbB3, anti-phospho-ErbB4 (Cell Signaling Technology), anti-Cofilin and anti-phospho-Cofilin (Novus Biological). The therapeutic antibody was not capable of recognizing denature antigen and therefore, could not be used for immunoblot analysis. Secondary antibodies were IRDye 680 conjugated goat anti-mouse IgG and IRDye 800 CW conjugated goat anti-rabbit IgG (Li-Cor Biosciences). Images were recorded and band intensities determined by LICOR.

### Fluorescence-activated cell sorting (FACS) analysis

293-NRG1-NTMyc cells were treated with anti-ragweed or anti-NRG1 antibodies for 72 hours. Cells were detached with 5 mM EDTA in PBS, washed with PBS and incubated with anti-Myc-Alexa Fluor 588 antibodies (Cell Signaling Technology) for 30 minutes on ice. FACS data was analyzed by FlowJo software.

### MYC ELISA

293 cells stably expressing NRG1-NTMyc cells were cultured in serial dilutions of antibodies for 72 hours. The washed cells were incubated with anti-Myc-HRP antibodies (1:800) (Cell Signaling Technology) for 1 hour at 4 °C. Cells were washed 3 times with washing buffer (PBS containing 1% FBS), and incubated with TMB for 15 minute. After adding stop reagent, the optical density was measured at 450/630 nm.

### Data availability statement

The datasets generated during and/or analyzed during the current study are available from the corresponding author on reasonable request.

### Statistical analysis

Data were analyzed and graphed using GraphPad Prism Software, San Diego California USA, using the indicated statistical tests.

## Electronic supplementary material

Supplementary Methods and Figures
