# Supplementary material for: Antibody-mediated stabilization of NRG1 induces behavioral and electrophysiological alterations in adult mice
Source: Sci Rep. 2018 May 29;8:8239. doi: 10.1038/s41598-018-26492-4 (PMC5974084; doi:10.1038/s41598-018-26492-4)
Supplement: Supplementary file 1 — Supplementary Methods and Figures [file 41598_2018_26492_MOESM1_ESM.docx]

**Antibody-mediated stabilization of NRG1 induces behavioral and electrophysiological alterations in adult mice**

**Sara L. Dominguez^1,+^, Ganapati V. Hegde ^2,8,+^, Jesse E. Hanson^1^, Hong Xiang^3^, Danielle Mandikian^3^, C. Andrew Boswell^3^, Cecilia Chiu^4^, Yan Wu^4^, Siao Ping Tsai^5^, Daniel Fleck^1^, Martin Weber^1^, Hai Ngu^6^, Kimberly Scearce-Levie^1,7^ and Erica L. Jackson^2,8^**

**SUPPLEMENTAL METHODS**

**Startle Chambers**

Startle chambers (SR-Lab, San Diego Instruments, San Diego, CA, USA) consisted of a Plexiglas cylinder 3.8 cm in inner diameter resting on a 12.5 x 20.5 cm Plexiglas frame inside a ventilated enclosure. Noise bursts and background noise were presented via a high-frequency speaker mounted ~26 cm above the base of the cylinder. Motion was detected from within the cylinder via a piezoelectric accelerometer mounted below the frame using a sampling rate of 1000Hz.

**Startle Response Calculations**

The startle response for each animal was defined as the average of the startle response during 65ms beginning at the onset of 120 dB stimulus across all *pulse-alone* and *prepulse +* *pulse* trials. PPI was then calculated for each animal according to the following formula:

100-((startle amplitude on *prepulse* trials + pulse trials/startle amplitude on *pulse-alone* trials) * 100) and data was collapsed across trials of the same stimulus type.

**Tissue harvest and preparation** **for Muscle Histology**

Mice were anesthetized with 2.5% tribromoethanol (0.5 ml/25 g body weight) and transcardially perfused with PBS. Brains were collected, flash frozen in liquid nitrogen. Gastrocnemius muscles were collected from both legs of each mouse and were fixed in 2% PFA for 2.5 hours and then cryoprotected in 30% sucrose.

**Neuromuscular junction imaging/analysis**

Digital images of immunofluorescent-labeled muscle sections on glass slides were acquired using the Ariol SL-50 whole slide scanning system (Leica Biosystems, Buffalo Grove, IL).  Nuclear, alpha-bungarotoxin and VChAT signals were captured using a dapi, fitc and texas red filter, respectively, at x100 magnification. Images were batch-processed using Metamorph (Molecular Devices). The motor end-plate as labeled by alpha-bungarotoxin was identified using an intensity threshold and morphological opening/closing with dilation. The VChAT signal area within each end-plate identified was quantified using boolean logic and a local threshold. The VChAT signal area was normalized to the end-plate area to calculate a colocalization ratio for each end-plate.  For each animal, 9-12 serial muscle sections were analyzed and the percentage of total end-plates that had a colocalization ratio between 0.7-1 were considered innervated.

*Muscle sectioning and sampling*

Gastrocnemius muscles used for NMJ staining were longitudinally cryosectioned at 20µm thickness and every other section was collected. Four series of 3 sections, spaced at 200 µm intervals across the entire muscle, were used for staining and scoring.

Gastrocnemius muscles used for muscle spindle staining were crossed sectioned at 60 µm thickness and every section was collected across 12 slides. 2 series of 4 sections, spaced at 720 µm intervals across the middle 6mm of the muscle, were used for staining and counting.

**Radio labeling of NRG1 with In-111**

*DOTA conjugation*

1 mg of NRG1 (R&D) was reconstituted in sodium borate buffered saline, pH 8.5 at 5 mg/mL. DOTA-NHS-ester (B280, Macrocyclics), diluted to 50 mg/mL in DMSO, was added at a 5:1 molar ratio to the NRG1 and incubated with gentle agitation at room temperature for 1 hour. The DOTA-conjugated NRG1 was purified on a NAP-5 column (17-0853-02, GE Healthcare) that was equilibrated with 0.3 M ammonium acetate buffer, pH 7 (Buffer A). DOTA-NRG1 was concentrated to 3mg/mL using a Centricon concentrator with a 10 kDa MWCO (Amicon, Millipore).

*Radiolabeling*

17 µL of DOTA-NRG1 (3 mg/mL in Buffer A) was reacted with 3 µL of In-111 (~1 mCi, Nordion) for 1 hour at 37 ᵒC with gentle agitation. To quench the reaction, 75 µL of Buffer A and 5 µL of 0.05 M EDTA was added to the reaction tube and incubated at RT for 5 min. The In-111-DOTA-NRG1 was purified on a NAP-column equilibrated with PBS.

**SUPPLEMENTAL FIGURES**

**S1**

**Supplemental Figure S1**. Treatment with anti-NRG1 does not affect body weight (p > 0.05) (n = 12 F/group). Treatment started 3 days before week 1 weights were taken. Data are shown as ± SEM.

**S2**

**Supplemental Figure S2**. Co-treatment with Anti-NRG1 but not anti-ErbB4/anti-ErbB3 affects motor behavior at week 1. **a.** Movement index measurements. ANOVA found a significant treatment effect (F (2,33) = 19.20, p < 0.0001. Post hoc comparisons (Tukey’s t-test) found significant increases in tremor-associated movement intensities between Anti-Ragweed and Anti-NRG1 (****p < 0.0001) but not between Anti-Ragweed and Anti-ErbB4/Anti-ErbB3 combo (n = 12 F/group). **b.** Number of open field rearings. ANOVA found a significant treatment effect (F (2,33) = 5.405, p = 0.0093). Post hoc comparisons (Tukey’s t-test) yielded significant decreases in number of rearings between Anti-Ragweed and Anti-NRG1 (*p < 0.05) but not between Anti-Ragweed and Anti-ErbB4/Anti-ErbB3 combo after 1 week on treatment (n = 12 F/group). All data shown as mean ± SEM.

**S3**

**Supplementary Figure S3.** Anti-NRG1 treatment does not inhibit ERBB signaling in hippocampus of wt mice. Western blot for lysates of hippocampus from wt mice treated with anti-NRG1.

**S4**

**Supplement Figure S4.**  Additional analysis of open field data from Figure 3A. Weeks 5 and 10 were averaged as “ on treatment” time points and weeks 14 and 18 were averaged as “recovery” time points. Unpaired t-tests for the average on treatment time point revealed significant effects between treatments (**p = 0.0067) for Anti-Ragweed vs. Anti-NRG1 but not for Anti-Ragweed vs. Anti-ErbB4 (p = 0.2369). Unpaired t-test for the average recovery time point also found no significant effects of treatment for either the Anti-Ragweed vs Anti-NRG1 (p = 0.1049) or the Anti-Ragweed vs. Anti-ErbB4 (p = 0.0589) (n = 7-11 F/group). The non-significant trend towards increased activity during recovery from anti-ErbB4 treatment is likely not meaningful given the lack of effect during ErbB4 treatment. The non-significant trend towards increased activity during recovery from anti-NRG1 treatment makes it hard to interpret whether recovery of normal activity levels was occurring. Data are shown as mean ± SEM.

**S5**

**Supplemental Figure S5.** Co-treatment with Anti-NRG1 but not anti-ErbB4/anti-ErbB3 affects % PPI at week one of dosing. **a.** One way ANOVA for Average % PPI yields an overall effect of treatment (F (2,33) = 5.245, p = 0.0105. Post hoc analysis using Unpaired t-test revealed no treatment effect of the Anti-ErbB4/Anti-ErbB3 when compared to Anti-Ragweed (p = 0.2596). While the anti-NRG1 treatment when compared to anti-Ragweed treatment does not reach significance in this study, there is a strong trend towards an Anti-NRG1 treatment effect (p = 0.0722), which is concordant with the results obtained in Figure 3b (week 1). There was a significant effect between the Anti-NRG1 vs. Anti-ErbB/Anti-ErbB3 treatment (**p = 0.0023). Data are shown as ± SEM.

In an additional analysis of the two data sets pooled together (fig.3b with fig S5), there was a significant effect of anti-NRG1 treatment when compared to anti-Ragweed treatment (p = 0.0002).

**S6**

**Supplemental Figure S6.** Anti-NRG1 causes accumulation of full length NRG1. **a.** Uncropped western blot for an experiment as shown in fig. 5 in HEK293 cells overexpressing NRG1 showing an increase in full length NRG1 upon treatment with anti-NRG1and an increase in the ratio of FL-NRG1 to NRG1-ICD. **b.** Western blot in HEK293 cells overexpressing NRG1 treated with a combination of pan-ADAM + BACE inhibitor showing an increase in FL-NRG1 and an increase in the ratio of FL-NRG1:NRG1-ICD. Data suggest that both anti-NRG1 and sheddase inhibitors interfere with processing of NRG1 in a similar fashion.
